# Associations between suppressive antibiotic therapy, treatment failure, and side effects among young, immunocompetent veterans with prosthetic joint infection who undergo debridement, antibiotics, and implant retention

**DOI:** 10.1017/ash.2025.10273

**Published:** 2026-02-23

**Authors:** Marin Leigh Schweizer, Rajeshwari Nair, Kelly Richardson Miell, James Merchant, Brice Beck, Bruce Alexander, Daniel Suh, Hiroyuki Suzuki, Aaron J. Tande, Mireia Puig-Asensio, Kimberly C. Dukes, Julia Walhof, Andrew Pugely, Ambar Haleem, Michael Scolarici, Poorani Sekar

**Affiliations:** 1 William S. Middleton VA Hospital, Madison, WI, USA; 2 Department of Medicine, School of Medicine and Public Health, University of Wisconsin-Madison, Madison, WI, USA; 3 Department of Internal Medicine, Carver College of Medicine, University of Iowa, Iowa City, IA, USA; 4 Center for Access and Delivery Research and Evaluation, Iowa City VA Health Care System, Iowa City, IA, USA; 5 Department of Biostatistics, College of Public Health, University of Iowa, Iowa City, IA, USA; 6 Division of Public Health, Infectious Diseases and Occupational Medicine, Department of Medicine, Mayo Clinic, Rochester, MN, USA; 7 Department of Infectious Diseases, Hospital Universitari de Bellvitge, Barcelona, Spain; 8 Centro de Investigación en Red de Enfermedades Infecciosas (CIBERINFEC; CB21/13/00009), Instituto de Salud Carlos III, Madrid, Spain; 9 Department of Orthopedics and Rehabilitation, Carver College of Medicine, University of Iowa, Iowa City, IA, USA

## Abstract

**Objective:**

Suppressive antibiotic therapy (SAT) is used to prevent recurrent prosthetic joint infections (PJI) among patients who undergo debridement, antibiotics, and implant retention (DAIR). We aimed to assess SAT outcomes among younger, immunocompetent patients.

**Design:**

Retrospective cohort study.

**Patients:**

Immunocompetent patients <65 years of age who received DAIR for PJI of the hip, knee, or shoulder.

**Setting:**

Veterans Affairs hospitals.

**Methods:**

SAT was divided into short-term (oral antibiotics given for <3 months after guideline concordant therapy) and long-term SAT (>3 months to 5 years of oral antibiotics). The primary outcome was treatment failure (TF) and mortality combined. SAT was a time-dependent covariate in Cox proportional hazards models.

**Results:**

Of the 938 patients, 15% received short-term SAT, 20% received long-term SAT, and 65% did not receive SAT. Short- and long-term SAT were significantly associated with decreased hazards of TF or mortality (short-term SAT adjusted hazard ratio (aHR) = 0.27; 95% confidence interval (CI): 0.11, 0.67; Long-term SAT aHR = 0.52; 95% CI: 0.30, 0.89). Short-term SAT was significantly associated with *C. difficile* infection (aHR: 3.47; 95% CI: 1.38, 8.74). Short-term SAT (aHR: 7.83; 95% CI: 4.80, 12.77) and long-term SAT (aHR: 1.68; 95% CI: 1.19, 2.38) were significantly associated with antibiotic-associated diarrhea. Long-term SAT was not significantly associated with TF alone (aHR = 0.61; 95% CI: 0.32, 1.16).

**Conclusions:**

SAT was significantly associated with decreased death or TF and increased side effects. Benefits and risks must be weighed before prescribing SAT to younger, immunocompetent patients.

## Introduction

Prosthetic joint infection (PJI) is a top reason for total joint arthroplasty failure, occurring after 0.5%–2% of total joint arthroplasties.^1–3^ PJIs are associated with high morbidity, mortality, and costs.^1^ In 2017, the annual U.S. hospital costs to treat PJIs was >$900 million.^4^ Models predict this will increase to $1.85 billion by 2030, with a 500% increase in total joint arthroplasties performed by 2,050.^4,5^

PJI treatment includes surgery and antibiotics. Debridement, antibiotics, and implant retention (DAIR) is a common treatment for PJI to salvage the infected prosthesis while minimizing morbidity and convalescence.^6^ This includes debridement followed by intravenous and/or highly bioavailable oral antibiotics.^6^

Long-term suppressive antibiotic therapy (SAT) is defined as the long-term use of oral antibiotics after initial PJI treatment.^7^ Long-term SAT is used to prevent PJI treatment failure (TF) and prevent or prolong the progression to further surgery, especially limb-threatening surgery.^8,9^ There is limited literature on the benefits of long-term SAT, and practices vary.^3,6,10–12^ The Infectious Disease Society of America (IDSA) guideline on PJI treatment lacks standardized guidance on who should receive long-term SAT after DAIR.^13^ This guideline mentioned that some panel members prescribe long-term SAT to older or immunosuppressed patients, and that long-term SAT in young patients is controversial.^13^ The frequency with which younger and immunocompetent patients receive long-term SAT, and whether it benefits them is unclear.

Long-term SAT overuse could lead to side effects including antibiotic-associated diarrhea (AAD) and *Clostridioides difficile* infection (CDI).^14^ Short-term SAT may be beneficial and may better align with antibiotic stewardship.^8^ However, a recent study among DAIR patients found that three months of oral antibiotics after intravenous antibiotics was associated with a higher risk of TF compared with one year.^15^

We aimed to assess the frequency of short-term and long-term SAT use among younger (ie, age <65 years), immunocompetent patients treated at any Veterans Affairs (VA) hospital. We also aimed to compare the incidence of TF, all-cause mortality, and antibiotic-associated side effects among those who did and did not receive short-term and long-term SAT.

## Methods

This retrospective cohort study included patients who received treatment for a hip, knee, or shoulder PJI at any VA hospital between January 1, 2003, and December 31, 2017. Patients were included if they received DAIR within 30 days before or six months after an International Classification of Diseases (ICD) code for PJI, and had a positive microbiology culture. DAIR was identified using CPT, ICD-9, and ICD-10 codes (Appendix 1). Cultures from hip, knee, shoulder, bone, joint, tissue, or body fluid were included.

**Figure 1.**
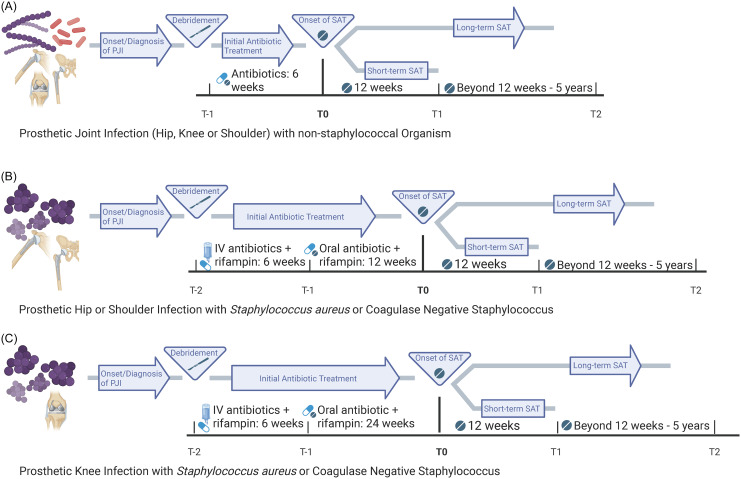
Timelines of antibiotic treatment by infected joint and pathogen. Footnote: t_0_ is when the time clock starts for antibiotic suppression. SAT, suppressive antibiotic therapy; IV, intravenous. Created in BioRender. Scolarici, M. (2025) https://BioRender.com/5gcyc2s.

Patients were excluded if they were ≥65 years of age, had an ICD code for an immunocompromising condition (eg, malignancy, organ transplantation, human immunodeficiency virus) or received a prescription for oral or parenteral immunosuppressive medications (eg, chemotherapeutic agents or steroids) within 30 days before the DAIR, or received anti-rejection medications within 90 days before the DAIR (Appendix 2). Patients were also excluded if they had three or more debridement operations on the same body site during a single admission, or if they had two-stage exchange surgery in the 30 days after the debridement, in order to exclude patients who we were unable to differentiate between initial treatment and TF. We excluded patients who had TF before time zero (Figure 1). Electronic medical record (EMR) review was performed on 527 patients who had an ICD code for PJI to evaluate them for inclusion and exclusion. EMR review optimized code selection to exclude orthopedic operations other than on the hip, knee or shoulder. Further EMR review on included patients validated data on joint infected, symptoms of PJI, DAIR procedure, clinician prescribing SAT, SAT dates, side effects, and non-PJI antibiotics.

We used medical, pharmaceutical, microbiological, and demographic data extracted from the VA EMR located in the VA Corporate Data Warehouse (CDW) and the Inpatient Main data set from the National Patient Care Database. Laboratory data were extracted from the CDW Microbiology 2.0 and LabChem datasets. These data were accessed through the VA Informatics and Computing Infrastructure.

This study was approved by the University of Iowa Institutional Review board and the Research and Development Committee of the Iowa City VA Health Care System. A waiver of informed consent was approved.

Long-term SAT was defined as more than three months of oral antibiotics after completion of guideline concordant therapy. Patients who received antibiotics within the three-month period after completion of guideline concordant therapy were categorized as receiving short-term SAT. All other patients were categorized as receiving no SAT. Guideline concordant therapy differed based on the joint infected and the infecting organism (Figure 1). For patients infected with a non-staphylococcal organism, guideline concordant therapy was defined as receipt of antibiotics for six weeks after DAIR (Figure 1A). For patients with a prosthetic hip or shoulder infection caused by staphylococcus species, guideline concordant therapy was defined as six weeks of intravenous antibiotics followed by oral antibiotics for three months (Figure 1B). For patients with prosthetic knee infections caused by staphylococcus species, guideline concordant therapy was defined as six weeks of intravenous antibiotics followed by oral antibiotics for six months (Figure 1C).^13,16^ To account for variation in the interpretation of the guidelines for staphylococcal infections and ensure that antibiotics classified as SAT were intended as SAT and not initial treatment, we defined SAT as beginning after 3 months (hip, shoulder) or 6 months (knee) of oral antibiotic therapy. SAT was determined to have ended if TF occurred, if the antibiotic was stopped and not restarted, or if there was gap in antibiotic use longer than two weeks.

The primary outcome of interest was TF or all-cause mortality that occurred up to 63 months after completion of initial antibiotic therapy (Figure 1). TF was defined as microbiologically confirmed PJI relapse or reinfection, or additional debridement, reoperation, or amputation at the same site. Secondary outcomes included TF alone, and antibiotic-associated side effects, including CDI and AAD. CDI were defined as a *C. difficile* positive lab assay or a prescription for oral vancomycin, fidaxomicin, or metronidazole within 72 hours of a lab assay for *C. difficile*. AAD was defined as a negative *C. difficile* lab assay result or by presence of an ICD code for AAD. For the primary outcome of interest, follow-up was censored at the end of the follow-up period or December 31, 2017, whichever came first. For secondary outcomes, follow-up was censored at death, the end of the follow-up period, or December 31, 2017, whichever came first. All outcome data were collected via the EMR (eg, VA CDW). Data validation was performed through manual chart review via VA Joint Legacy Viewer for a subset of patients.

Comorbidities described by Elixhauser and Charlson were measured using ICD codes from the year prior to PJI.^17^ The components of the modified Acute Physiology and Chronic Health Evaluation (APACHE) III score were used to assess patients’ severity of illness at the time of debridement.^18^ Erythrocyte sedimentation rate (ESR) and C-reactive protein (CRP) laboratory values were measured within 10 days of DAIR.

Descriptive statistics were calculated to describe the demographic, clinical, and microbial characteristics of the patients and to characterize the occurrence of adverse events, categorized by antibiotic status (long-term or short-term SAT and no SAT). Chi-square tests and two-sample t-tests were used to assess bivariable associations.

**Figure 2.**
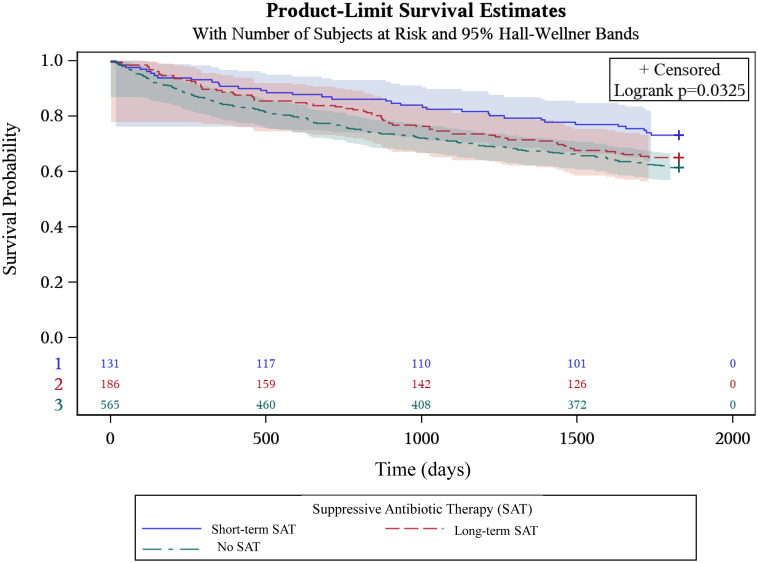
Kaplan-Meier curve of time to treatment failure among patients who did not die or experience treatment failure in the first three months after guideline concordant treatment. Comparison of long-term suppressive antibiotic therapy (SAT) (red dotted line) with short-term SAT (blue solid line) and no SAT (green dotted line). The *P* value refers to a three-way comparison between short-term SAT, long-term SAT and no SAT. Footnote: Time is measured in days. Shaded regions are 95% Hall-Wellner Bands.

Cox proportional hazards models were created using SAT as a time-dependent covariate and short-term SAT as a categorical time-dependent interaction term. An indicator for SAT was created by putting the data in start-stop format, with a value of 1 when on antibiotic suppression and 0 when off. To capture the time period effect SAT, the data were put in start-stop format a second time, with a single cut-point at 3 months.^19^ From time 0 to 3 months, a patient was considered to be in the short-term SAT period, and from 3 months to stopping antibiotics or the end of the study period, a patient was considered to be in the long-term SAT period. Backward selection with a criterion of *P* < .20 was employed in model building. Long-term SAT, short-term SAT, infection site, and infecting organism (staphylococci vs other) were forced into the model regardless of statistical significance. To assess the impact of immortal time bias, we performed a subset analysis of patients who survived and did not experience TF in the first three months after initial treatment and compared short-term SAT, long-term SAT, and no SAT (Figure 2). An exploratory analysis compared one year of SAT with more than one year of SAT and no SAT.

Data cleaning and residual analysis were performed using R version 4.1.2. Variable selection, model fitting, and plotting were performed using SAS 9.4.^20^ Power calculations showed sufficient power (>80%) to see a statistically significant difference in TF (*α* = 0.05).

## Results

In total, 938 patients from 104 VA medical centers met the inclusion criteria. The median age was 58 years, and most patients were white males (Table 1). Nearly two-thirds (64%) had prosthetic knee infections, 28% had prosthetic hip infections, and 8% had prosthetic shoulder infections. Overall, 81% had a PJI caused by staphylococci, 10% had a PJI caused by other Gram-positive organisms (ie, enterococci, streptococci, *C. acnes*), 5% had a PJI caused by Gram-negative organisms, and 4% had polymicrobial infections.

**Table 1.** Characteristics of the study cohort by receipt of suppressive antibiotic therapy after initial PJI treatment

Characteristic	Long-term suppressive antibiotic therapy (SAT) for >3 months (N = 187)	Short-term SAT (≤3 months after initial PJI treatment) (N = 139)	No SAT after initial PJI treatment (N = 612)	Total (N = 938)
**Male sex**	184 (98.4%)	132 (95.0%)	580 (94.8%)	896 (95.5%)
**Age (years) at debridement, mean**	56.6	58.0	55.7	56.2
**Race** ^a^				
Black	37 (19.8%)	24 (17.1%)	141 (23.1%)	202 (21.5%)
White	140 (74.9%)	103 (74.1%)	432 (70.6%)	675 (72.0%)
Other	5 (2.7%)	7 (5.0%)	17 (2.8%)	29 (3.1%)
**BMI (kg/m^2^** **), mean** ^b^	32.0	32.1	30.6	31.1
**Weight categories** ^b^				
Underweight	3 (1.7%)	1 (0.7%)	2 (0.3%)	6 (0.7%)
Normal	22 (12.2%)	16 (11.5%)	141 (24.1%)	179 (19.8%)
Overweight	47 (26.1%)	45 (32.4%)	164 (28.0%)	256 (28.3%)
Obese	108 (60.0%)	76 (55.1%)	280 (47.7%)	464 (51.3%)
**Joint involved**				
Hip	53 (28.3%)	38 (27.1%)	170 (27.8%)	261 (27.8%)
Knee	124 (66.3%)	91 (65.5%)	387 (63.2%)	602 (64.2%)
Shoulder	10 (5.3%)	10 (7.1%)	55 (9.0%)	75 (8.0%)
**Organism causing PJI**				
*Staphylococcus* species	135 (72.2%)	94 (67.9%)	534 (87.2%)	763 (81.3%)
*Streptococcus* species	25 (13.4%)	13 (9.3%)	34 (5.6%)	72 (7.7%)
*Enterococcus* species	0 (0.0%)	2 (1.4%)	7 (1.1%)	9 (1.0%)
*C. acnes*	7 (3.7%)	2 (1.4%)	7 (1.1%)	16 (1.7%)
*Pseudomonas* species	1 (0.5%)	5 (3.6%)	1 (0.2%)	7 (0.7%)
Other gram-negative organisms	8 (4.3%)	20 (14.3%)	10 (1.6%)	38 (4.1%)
Polymicrobial infection	11 (5.9%)	3 (2.1%)	19 (3.1%)	33 (3.5%)
**Comorbidities**				
Coagulopathy	7 (3.7%)	5 3.6%)	29 (4.7%)	41 (4.4%)
Alcohol abuse	25 (13.4%)	17 (12.2%)	90 (14.7%)	132 (14.1%)
Renal disease	9 (4.8%)	5 (3.6%)	36 (5.9%)	50 (5.3%)
Dialysis	3 (1.6%)	2 (1.4%)	10 (1.6%)	15 (1.6%)
Liver disease	16 (8.6%)	6 (4.3%)	45 (7.4%)	67 (7.1%)
Severe liver disease	3 (1.6%)	2 (1.4%)	9 (1.5%)	14 (1.5%)
Drug abuse	24 (12.8%)	8 (5.7%)	62 (10.1%)	94 (10.0%)
Paralysis	1 (0.5%)	3 (2.2%)	11 (1.8%)	15 (1.6%)
**Severity of illness (modified APACHE III), mean** ^c^	27.19	27.30	28.20	27.87
**Laboratory findings at time of DAIR**				
CRP, mg/dL, mean^d^	8.77	7.13	7.04	7.41
ESR, mm/h, mean^e^	42.76	46.90	44.23	44.33
**Facility complexity** ^f^				
1a (Highest)	18 (9.8%)	29 (21.2%)	86 (14.4%)	133 (14.5%)
1b	38 (20.8%)	24 (17.4%)	118 (19.7%)	180 (19.6%)
1c	115 (62.8%)	79 (57.2%)	367 (61.4%)	561 (61.0%)
2	11 (6.0%)	4 (2.9%)	27 (4.5%)	42 (4.6%)
3 (Lowest)	1 (0.5%)	1 (0.7%)	1 (0.2%)	3 (0.3%)

Footnote: PJI, prosthetic joint infection; ESR, Erythrocyte Sedimentation Rate within 10 days of DAIR; CRP C-Reactive Protein within 10 days of DAIR; ^a^32 missing values; ^b^33 missing values; ^c^47 missing values; ^d^38 missing values; ^e^191 missing values; ^f^19 missing values.

Fifteen percent (n = 139) of patients received some antibiotics in the three months after initial treatment for the PJI (ie, short-term SAT), 20% received antibiotics for over three months after initial treatment for the PJI (ie, long-term SAT), and 65% did not receive any antibiotics after initial treatment for the PJI. The median duration of antibiotics after guideline concordant therapy in the short-term SAT group was 34 days and in the long-term SAT group was 268 days.

Compared with those who received short-term SAT or no SAT, those who received long-term SAT were more likely to have a PJI caused by *C. acnes,* streptococci, or a polymicrobial infection, and to be obese. Those who received short-term SAT were significantly older and more likely to receive debridement at a higher complexity facility than those who received long-term SAT or no SAT (Table 1). There were no statistically significant differences in comorbidities or severity of illness at time of debridement between groups.

Overall, 26.4% of patients had TF and 19.7% died during the follow-up period. The incidence of TF did not differ by infecting organism or infection site. 21.9% of patients who received long-term SAT experienced TF compared with 22.3% who received short-term SAT, and 28.8% of patients who did not receive additional antibiotics (*P* = .09; Table 2). There was no significant difference in TF when comparing those on short-term SAT with those on long-term SAT (*P* = .47). The median time to TF among those who did not receive additional antibiotics was 345 days. The median times to TF were 410 days for short-term SAT and 432 days for long-term SAT (*P* = .19). The median time to TF among those who failed while on SAT was 130 days. Of those who stopped SAT before TF, the median time from stopping SAT to TF was 414 days. All-cause mortality was significantly higher among patients who did not receive SAT (22.2%) compared with those who received long-term SAT (15.5%) or short-term SAT (14.4%; *P* = .03).

**Table 2.** Outcomes associated with chronic suppressive antibiotic therapy (SAT), short-term SAT and no antibiotics after initial PJI treatment

	Long-term SAT for >3 months (N = 187)	Short-term SAT (≤3 months after initial PJI treatment) (N = 139)	No SAT after initial PJI treatment (N = 612)	Total (N = 938)	*p*-value
**Any treatment failure**	41 (21.9%)	31 (22.3%)	176 (28.8%)	248 (26.4%)	.087
**All-cause mortality**	29 (15.5%)	20 (14.4%)	136 (22.2%)	185 (19.7%)	.030
***C. difficile* Infection**	4 (2.1%)	5 (3.6%)	8 (1.3%)	17 (1.8%)	.176
**Antibiotic- associated diarrhea**	26 (13.9%)	25 (18.0%)	92 (15.0%)	143 (15.2%)	.580

Receipt of short-term SAT was significantly associated with decreased hazards of the composite outcome of TF or death (adjusted hazard ratio (aHR) = 0.27; 95% CI: 0.11, 0.67) after adjusting for infecting organism, infection site, comorbidities, and obesity. Similarly, short-term SAT was associated with decreased hazards of TF alone (aHR = 0.24; 95% CI: 0.09, 0.68).

Receipt of long-term SAT was significantly associated with decreased hazards of the composite outcome of TF or death (aHR: 0.52; 95% CI: 0.30, 0.89) after statistically adjusting for infecting organism, infection site, comorbidities, and obesity. However, receipt of long-term SAT was not statistically significantly associated with decreased hazards of TF (aHR = 0.61; 95% CI: 0.32, 1.16). In the subset of patients who survived and did not experience TF in the first three months after initial treatment, similar trends were seen in the association between short-term SAT, long-term SAT, and no SAT and TF (*P* = .033; Figure 2). However, short-term SAT and long-term SAT were not statistically significantly different (*P* = .27).

SAT was not significantly associated with side effects in the unadjusted analysis (Table 2). Overall, 17 patients developed a CDI, and this did not statistically differ by receipt of SAT in the unadjusted analysis (*P* = .17). Among the five patients who received short-term SAT, two stopped SAT within a week of their *C. difficile* test. Long-term SAT was not statistically significantly associated with CDI in the adjusted analysis (aHR for CDI: 1.42; 95% CI: 0.70, 2.87). However, short-term SAT was significantly associated with CDI (aHR: 3.47; 95% CI: 1.38, 8.74) after statistically adjusting for infection site, infecting organism, and comorbidities. Both short-term SAT (aHR: 7.83; 95% CI 4.80, 12.77) and long-term SAT (aHR: 1.68; 95% CI: 1.19, 2.38) were significantly associated with AAD when compared with no SAT after statistically adjusting for infection site, infecting organism, and comorbidities.

In an exploratory analysis, one year of SAT was statistically significantly protective compared with no SAT (adjusted HR = 0.33; 95% CI: 0.21, 0.53). However, there was not a statistically significant difference when comparing one year of SAT with more than one year of SAT (adjusted HR = 0.60; 95% CI: 0.28, 1.28).

## Discussion

In this multicenter retrospective cohort study of immunocompetent patients under the age of 65 years, 20% received long-term SAT, and 15% received short-term SAT. Both were associated with decreased risk of the composite outcome of death and TF but also with antibiotic-related side effects. The beneficial effects of SAT were stronger among those who received short-term SAT than those who received long-term SAT. This study may provide evidence for both extending initial PJI treatment and shortening SAT duration, rather than prescribing SAT indefinitely.

Our findings are similar to a multicenter study of SAT use among patients with total knee arthroplasty PJI.^8^ They found finite benefits of SAT from 6 weeks to one year after debridement and no benefit after one year.^8^ Another study comparing one versus five years of SAT in PJI patients treated with DAIR found no difference in TF.^15^ The DATIPO trial randomized patients with PJI to 6 versus 12 weeks of treatment and found that 6 weeks was not non-inferior to 12 weeks among patients that underwent DAIR.^21^ In our study, the subgroup of patients with non-staphylococcal PJI reflect this 6 week versus longer result and may have contributed to the overall benefit of SAT.

In our cohort, TF incidence was similar to that described in a systematic literature review of patients with PJI treated with DAIR and long-term SAT, which found that 25% of patients experienced TF.^6^ In our cohort of young, immunocompetent patients who were treated with DAIR and long-term SAT, 21.9% experienced TF.^6^ However, that review defined long-term SAT as antibiotic suppression for beyond 1 year (mean duration 18–63 months), whereas we defined it as 3 months after completion of guideline concordant therapy (median duration of long-term SAT was 21 months).^6^ That systematic review and another multicenter cohort study found that 15.4% to 26.8% of DAIR patients treated with long-term SAT experienced antibiotic-related side effects.^6,22^ In our cohort, 13.9% of patients who received long-term SAT experienced AAD and 2.1% had CDI. We likely underestimated the true effect of SAT on side effects as non-gastrointestinal side effects were not assessed.

Surprisingly, patients who received short-term SAT had a higher incidence of side effects compared with those who received long-term SAT. Early side effects may have influenced SAT continuation decisions. Additionally, patients at high risk for antibiotic-associated side effects (eg, older age) may have been less likely to receive long-term SAT. This difference may also reflect variation in antibiotics prescribed for short-term versus long-term SAT or differences in gut microbiome dysregulation in these time periods.

Prior SAT studies used heterogenous patient cohorts, and few accounted for immunosuppression or age. Our study evaluated only younger, immunocompetent patients who underwent DAIR. Prior studies also did not account for antibiotic prescribing practices that differ by pathogen and body site of infection.^13,16^ We accounted for pathogen and infection site when determining when to measure SAT (Figure 1). A recent review article stated that only three studies adequately differentiated SAT from initial PJI treatment.^23^ We used a conservative approach to defining SAT to avoid misclassifying antibiotics meant to be initial staphylococcal PJI treatment, since some physicians interpret guideline recommendations differently. We allowed for a full 3- or 6-month duration of oral antibiotics after the 6-week intravenous antibiotic treatment period, rather than including the intravenous antibiotics in the 3- or 6-month time frame (Figure 1).

This study had limitations. Treatment strategies were not standardized and physicians’ prescribing patterns and skill level vary, potentially causing unmeasured confounding. Similarly, we were unable to ascertain the providers’ intent and whether long-term SAT was interrupted due to TF or side effects. This would have biased our results toward the null. We also could not assess guideline concordant intravenous antibiotic completion prior to SAT due to inability to track home infusion companies. We could not determine whether initial PJIs were acute or chronic infections. However, a recent systematic literature review did not find a difference between acute and chronic infections in terms of the association between long-term SAT and outcomes.^6^ We also could not measure additional risk factors including late acute versus early postoperative PJI, polyethylene/modular component exchange, or whether DAIR was performed arthroscopically. We may have overestimated the number of patients with clinically significant CDI because our definition did not require multiple positive tests or *C. difficile* treatment. Finally, we included patients treated between 2003–2017 and PJI treatment strategies have changed based on recent data (eg, DATIPO trial).^21^

In conclusion, our multicenter cohort study of young, immunocompetent patients found an association between SAT and reduced TF or mortality, with the strongest effect among those who received short-term SAT. These data support a time-limited use of SAT. In the absence of a randomized trial, providers and patients should discuss the benefits and harms of SAT to make a shared decision, with periodic reassessment.
